# Structural characteristics, multifunctional applications, and research prospects of ferritin: a case study of sturgeon ferritin

**DOI:** 10.3389/fnut.2025.1656213

**Published:** 2025-07-23

**Authors:** Nida Maqsood, Zuoliang Tang, Abu Zar Ghafoor, Shuhong Li, Zhiqing Zhang, Anjun Chen, Zhiyong Zou, Man Zhou, Meiliang Li

**Affiliations:** ^1^College of Food Science, Sichuan Agricultural University, Ya’an, China; ^2^College of Mechanical and Electrical Engineering, Sichuan Agricultural University, Ya’an, China; ^3^Department of Biometry, Institute of Agriculture, Warsaw University of Life Sciences, Warsaw, Poland

**Keywords:** ferritin, fresh-cut fruit preservation, food nutrition, sturgeon ferritin, nanocarrier, food waste

## Abstract

Ferritin is a ubiquitous cage-shaped protein found in living organisms. Beyond its fundamental role in iron homeostasis, ferritin demonstrates growing application value in food engineering, nanocarrier systems, and biomedicine, owing to its unique self-assembly properties, exceptional stability, and biocompatibility. This review systematically summarizes the core structural features and physicochemical properties of ferritin, with a particular focus on its applications across three major domains. In food engineering, ferritin acts as both a carrier for bioactive compounds and a highly efficient, low-irritant iron fortificant, significantly enhancing nutrient stability, solubility, and bioavailability, thereby extending food shelf life. In the nanocarrier field, its nanocage structure provides an ideal platform for constructing nutrient and drug delivery systems, enabling targeted transport and controlled release. In biomedicine, ferritin is utilized in tumor imaging, targeted therapy, and inflammation biomarker detection. Using sturgeon liver ferritin as a specific example, this review details its unique advantages derived from its source, such as distinctive structure, enhanced stability, and application potential. Furthermore, the review identifies key challenges in ferritin research, including structural variability, digestive stability, and long-term safety concerns. It also outlines future research directions, highlighting the immense potential of ferritin in addressing critical challenges like fresh-cut food preservation. With advancing technology and multidisciplinary integration, ferritin is poised to become a powerful interdisciplinary tool.

## 1 Introduction

**Figure 1:**
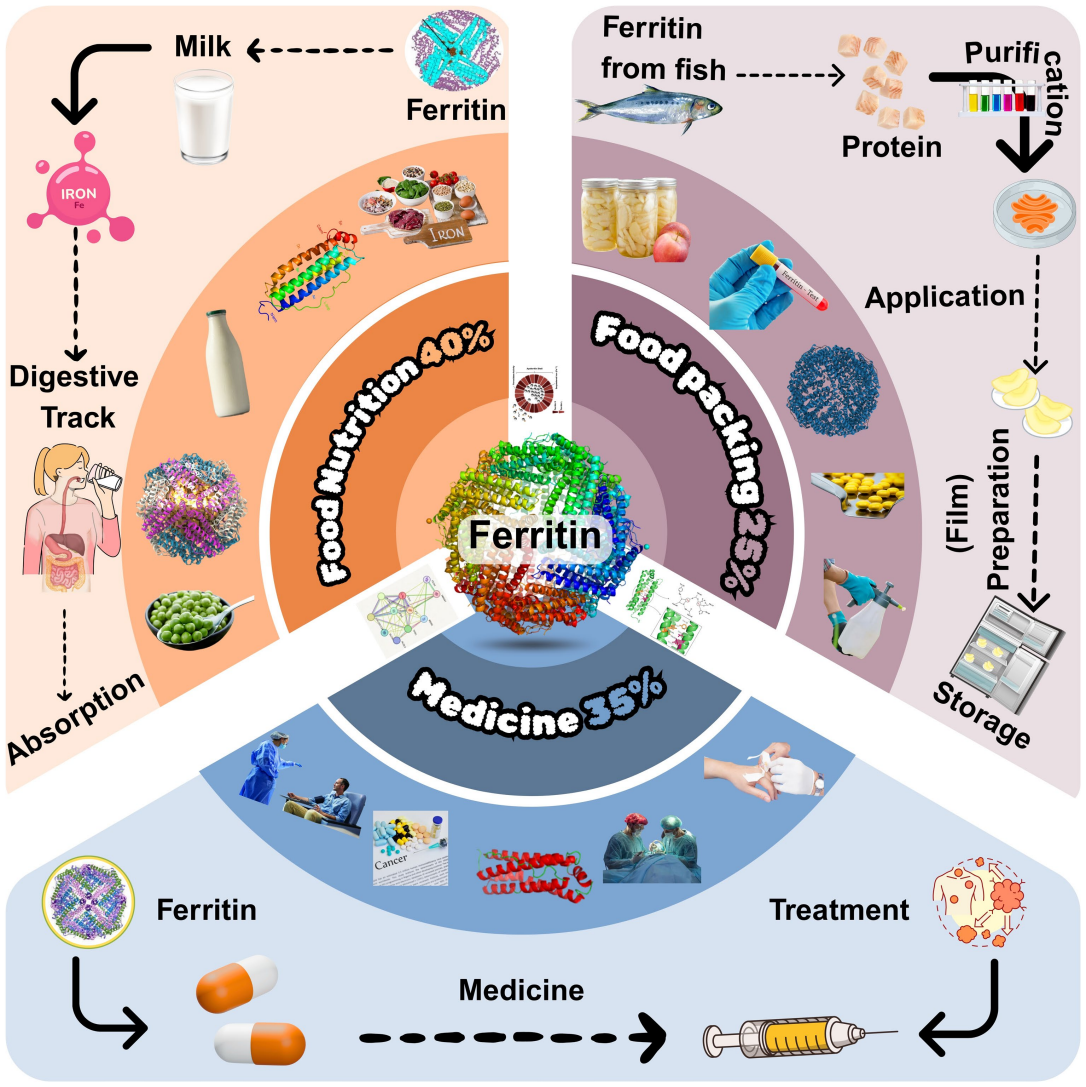
Distribution of ferritin applications across key industries.

In recent years, ferritin has gained significant attention across nutrition, food science, medicine, chemistry, and materials science. As a ubiquitous protein, ferritin not only stores iron but also functions in detoxification (1). Its core role involves sequestering iron ions and maintaining iron homeostasis. The application value of ferritin in food engineering, nanocarrier systems, and biomedicine has become increasingly prominent, particularly for addressing challenges such as food preservation, nutrient delivery, and disease diagnosis and treatment. As illustrated in Figure 1, this review focuses on the structural characteristics and multifunctional applications of ferritin with emphasis on shelf-life extension and explores its unique value and prospects using sturgeon ferritin as a representative model.

Ferritin, a cage-shaped protein complex ubiquitous in living organisms, features a unique quaternary structure that evolved early in life as an ancient solution for regulating iron homeostasis (2). Since its initial isolation from horse spleen by Laufberger (3), this nanoscale (12 nm outer diameter) protein cage self-assembled from 24 subunits has transcended its role as a mere iron-storage molecule. Its exceptional iron-sequestering capacity (up to 4,500 iron atoms per molecule), dynamic reversible assembly, and remarkable biocompatibility have positioned it as a frontier platform for interdisciplinary research (4). Traditionally, ferritin’s primary function was to chelate intracellular free iron, preventing Fenton reaction-induced oxidative damage while acting as an iron reservoir for precise release in response to metabolic demands (5). However, research over the past two decades has revolutionized its applications: the integration of nanotechnology and structural biology has unlocked ferritin’s disruptive potential in food engineering, nanomedicine, and biomaterials. This stems from its programmable nanocavity (8 nm inner diameter), pH-responsive assembly/disassembly behavior, and surface modifiability (6).

In food science, global food waste due to nutrient loss and spoilage reaches 1.3 billion tons annually (Food and Agriculture Organization of the United Nations), with fresh-cut fruits facing acute challenges like enzymatic browning, microbial invasion, and nutrient degradation triggered by mechanical damage (7). Conventional preservation techniques (e.g., chemical inhibitors, modified atmosphere packaging) face limitations in safety and functionality. Ferritin offers a novel solution: its antioxidant amino acid residues (e.g., glutamate, phenylalanine) scavenge free radicals; the nanocage encapsulates browning inhibitors (e.g., ascorbic acid, polyphenols); and its Pickering emulsion coatings modulate water activity and gas permeability (8, 9). Recent studies demonstrate that ferritin-based nanoencapsulation significantly enhances the quality of freeze-dried fruits and vegetables. For instance, apple slices exhibit 35–45% extended shelf life, >40% reduction in surface browning index, and 85–92% retention of vitamin C (10). These properties establish ferritin as a strategic tool against quality deterioration in fresh-cut produce.

The nanocarrier field benefits from ferritin’s precise loading and controlled release capabilities. Unlike synthetic nanomaterials with potential biotoxicity, ferritin is an endogenous protein with inherent biosafety. Its triple-axis channels (3–5 Å pore size) permit diffusion of small molecules, while pH-mediated subunit dissociation/reassembly enables efficient encapsulation of hydrophobic bioactives (e.g., curcumin, *β*-carotene), boosting their water solubility by 3-fold and photostability by 60% (11, 12). Notably, genetic engineering allows surface conjugation of targeting ligands (e.g., RGD peptides), enabling gut-directed nutrient delivery and advancing functional food design (13).

Biomedical applications leverage ferritin’s diagnostic and therapeutic value. Serum ferritin levels are established biomarkers of inflammation and cancer, with altered glycosylation patterns correlating strongly with early-stage hepatocellular and pancreatic carcinomas (14). Ferritin nanocages can also load contrast agents (e.g., Gd^3+^) or anticancer drugs, exploiting the enhanced permeability and retention (EPR) effect to target tumor tissues. In murine models, this strategy increases intratumoral drug concentration 5-fold while reducing systemic toxicity (15). Such “smart delivery” positions ferritin as an ideal platform for theranostics.

Despite its promise, functional heterogeneity arising from species-dependent variations remains unresolved. Ferritins from different sources exhibit significant differences in subunit composition (H/L ratio), channel structure, and stability. For example, the H-2 subunit (28 kDa) in plant ferritin (e.g., soybean) confers strong protease resistance, while mammalian ferritin (21 kDa heavy chain/19 kDa light chain) harbors a more defined ferroxidase center (16). Within this context, sturgeon liver ferritin (*Acipenser baerii* Ferritin) exemplifies unique advantages among aquatic sources: Fourier-transform infrared spectroscopy (FTIR) confirms its high *α*-helix content (72%), structural stability within pH 3.4–10 and 60–80°C, and capacity to store 4,500 iron atoms per molecule as a unique iron oxide/hydroxide core (with molecular mass of 474 kDa) (17–19). These properties make it a prime candidate for acidic food systems and low-temperature processing, particularly for preservation and nutrient fortification.

This review systematically analyzes ferritin’s structure function relationships, highlighting breakthroughs in food shelf-life extension (especially fresh-cut fruit preservation), nanocarrier engineering, and biomedical applications. Using sturgeon liver ferritin as a paradigm, we elucidate how source-specific ferritins enhance efficacy. We also address critical challenges controversies in iron bioavailability, insufficient digestive stability, and undefined long-term safety and propose targeted future directions: multiscale modeling of ferritin’s molecular interactions on fresh-cut fruit surfaces; development of sturgeon-ferritin-based smart coatings; and establishment of interdisciplinary platforms to accelerate clinical translation. This work aims to provide a theoretical foundation and technical roadmap for innovative ferritin applications in the food industry.

## 2 Structure and physicochemical properties of ferritin

**Figure 2:**
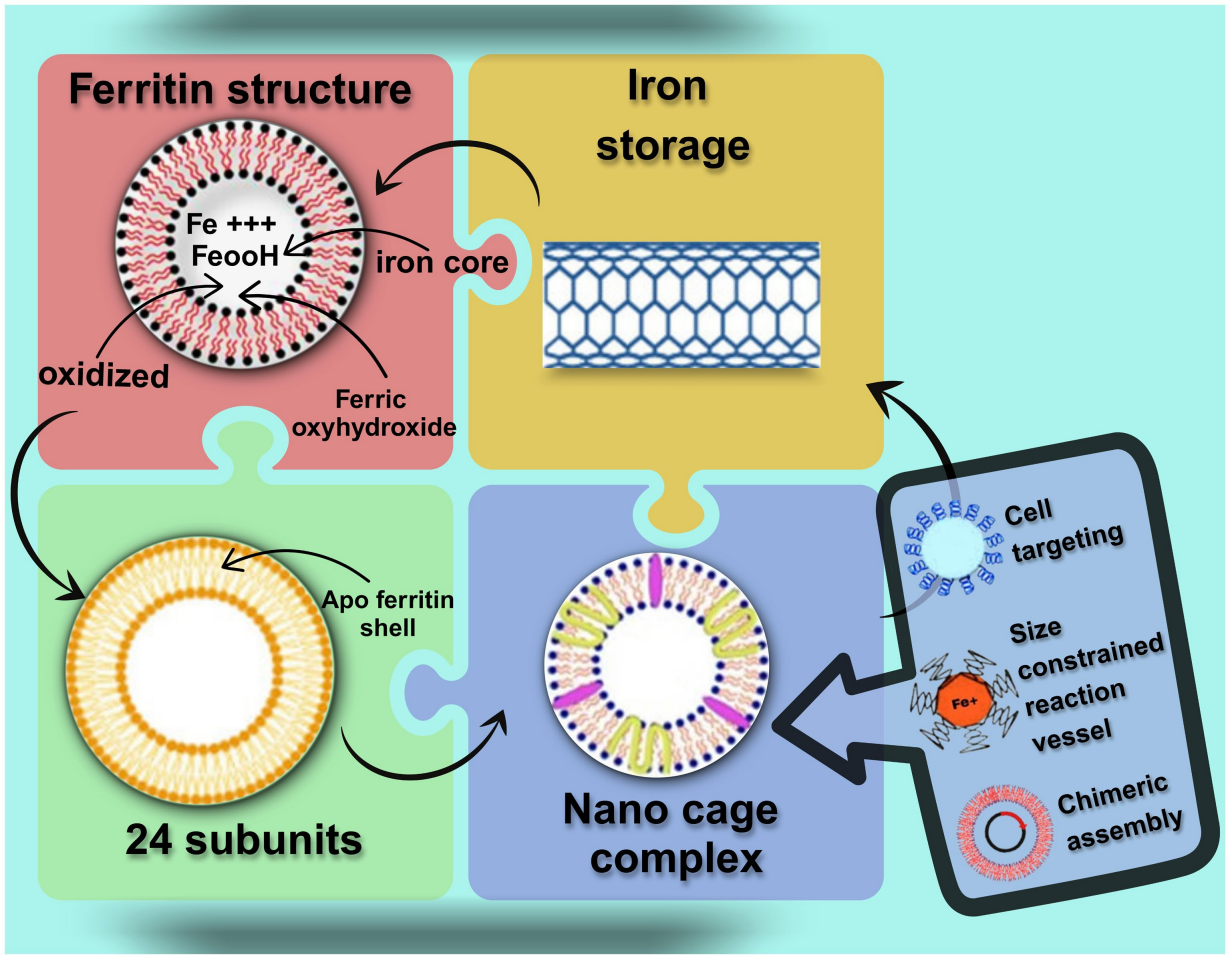
Ferritin molecular structure and iron storage.

Ferritin is an intracellular microcage structure. As depicted in Figure 2, it self-assembles from 24 subunits into a cage-like architecture with 4–3-2 symmetry, featuring an 8 nm inner cavity and a 12 nm outer diameter as described in Table 1. This structure can accommodate up to 4,500 iron atoms, positioning ferritin as a reliable and efficient iron-supplementing functional agent (20). Molecularly, ferritin selectively chelates excess intracellular iron ions and releases them precisely in response to metabolic demands, thereby maintaining iron homeostasis in a soluble, non-toxic, and bioavailable form. Notably, this iron-regulatory function is highly conserved across humans, plants, and other organisms, with its specific structural features establishing it as a critical iron storage and detoxification system (21, 22). Functionally, ferritin extends beyond iron storage: (i) it directs iron molecules to targeted intracellular sites via specific localization; (ii) its nanoscale cavity provides a precise and secure microenvironment for redox reactions; and (iii) its modular assembly enables participation in diverse cellular physiological activities.

**Table 1:** Ferritin structure–function analysis.

Structural feature	Description	Function	References
24-subunit assembly	Creates a symmetrical 4–3-2 structure with an outer diameter of 12 nm and an inner diameter of 8 nm.	Encapsulates bioactive compounds and produces a nano-cage for iron storage that can hold up to 4,500 iron atoms.	(8, 20)
Inner cavity	Under neutral conditions, the inner surface’s acidic residues (Glu and Asp) provide a negative charge density.	Provides an appropriate environment for encasing bioactive chemicals through electrostatic interactions; binds and stores iron ions in a redox-inactive state.	(85)
Pores (triple-axis and quadruple-axis channels)	Pore diameter vary between around 3 and 5 Å.	permits the entry and escape of tiny molecules during the encapsulation or release process, including iron ions and maybe some bioactive substances.	(85)
Ferroxidase site	Composed of conserved amino acid ligands (e.g., Glu27, Tyr34, Glu62, His65, Glu107, and Gln141 in the H-type subunit)	Helps in the oxidation of ferrous ions, which is essential for the ferritin core’s ability to store iron.	(86)

### 2.1 Structural details and assembly mechanism

Structurally, 12 pairs of ferritin subunits group in antiparallel orientations to form a regular dodecahedron with 4–3-2 axial symmetry. Each cylindrical subunit (5 nm length × 2.5 nm diameter) contains four *α*-helical bundles (antiparallel A–B and C–D pairs) and a shorter C-terminal α-helix (E). Helix E forms a 60° angle with the B–C helices, connected via an 18-residue non-helical BC loop. Surface charge distribution is distinctive: at neutral pH, the inner surface exhibits high negative charge density due to acidic residues (e.g., Glu, Asp), while the outer surface (comprising subunit N-termini, BC loops, and A/C helices) carries a slight positive or neutral net charge. Critically, each ferritin molecule contains eight threefold-axis channels and six fourfold-axis channels (3–5 Å pore diameter), forming a network that connects the protein cavity to the external environment (2, 23–25). This unique cage architecture allows cells to precisely regulate iron ion supply.

Regarding subunit composition, vertebrate ferritin primarily comprises heavy (H-type) and light (L-type) chains. Plant-derived ferritin exhibits distinct subunit characteristics: its light chains differ significantly from homopolymeric heavy chains, and molecular weights vary across sources. For example, soybean seed ferritin subunits are 26.5 kDa (H-1) and 28.0 kDa (H-2), markedly larger than their vertebrate counterparts (19.0 kDa light chain; 21.0 kDa heavy chain) (26). Mammalian ferritin typically consists of ~21 kDa heavy (H) and ~19.5 kDa light (L) chains, sharing 55% amino acid sequence similarity. The H-subunit harbors a binuclear ferroxidase center with conserved iron-binding sites (Glu27, Tyr34, Glu62, His65, Glu107, Gln141) responsible for rapid Fe^2+^ oxidation. As a prototypical pore protein, ferritin possesses six fourfold-, eight threefold-, and 12 twofold-axis channels (0.3–0.5 nm pore diameter) (2).

Ferritin’s reversible disassembly/reassembly driven by pH facilitates efficient encapsulation of bioactives. Its thermostability enables cost-effective production and purification in *Escherichia coli*, outperforming other nanocarriers. Under extreme acidity or alkalinity (pH ≤ 2.0 or ≥ 11.0), the cage disassembles into subunits; reassembly occurs at neutral pH. Composed of non-toxic elements, ferritin rarely elicits strong non-self-antibody responses. Its ferroxidase site (nucleation center) binds iron and other metal ions and can be functionally modified via chemical or genetic conjugation (27–30). This approach efficiently encapsulates molecules too large to diffuse through intact cage pores. Leveraging reversible self-assembly, numerous nutrients, drugs, and functional molecules have been successfully loaded into ferritin nanocages (30–34).

### 2.2 Species variability and subcellular localization

Ferritin, a multifunctional protein ubiquitous in bacteria, plants, and animals, has been extensively studied for its iron storage and detoxification roles (2). Structural studies reveal that >90% of iron in legume seeds is stored as ferritin (35). Compared to prokaryotes (which contain heme-bearing bacterioferritins, BFRs, and canonical H-type ferritins) and vertebrates, plant ferritin (phytoferritin) displays unique traits. Subcellularly, animal ferritin localizes to the cytoplasm, with expression tightly regulated by iron response elements (IREs) and iron regulatory proteins (IRPs) (36). In contrast, plant ferritin resides in plastids and is primarily transcriptionally regulated (37, 38). Although primary amino acid sequences lack homology, secondary, tertiary, and quaternary structures show significant conservation, indicating evolutionary preservation. In apoferritin cages, light subunits dominate (80–90%), while heavy subunits comprise 10–20%; sequence homology between them is 55%. Hydrophilic channel sequences are identical, but outer surface, cavity, and hydrophobic channel sequences differ (39, 40).

Ferritin source selection depends on application requirements. Plant-derived ferritin, with unique L-subunits and functional properties, excels in specific contexts. Animal-derived ferritin, with well-defined structure and established iron storage function, holds promise for biomedicine and therapy. Vertebrate-derived ferritin, with clear H/L subunit differentiation and high sequence similarity (55%), provides an ideal model for research and therapeutic development (41). Optimal ferritin types should be selected based on functional needs, as sources offer distinct advantages.

### 2.3 Characteristics of sturgeon liver ferritin

Sturgeon liver ferritin, a biomacromolecule with unique spatial conformation, demonstrates significant value in food science, biomedicine, and nanotechnology. Its 24 subunits self-assemble into a spherical nanocage (~12 nm diameter; ~8 nm cavity) as defined in Table 1, with highly symmetric architecture observable via transmission electron microscopy (TEM). Subunits are classified as H-type (≈20.5–21.1 kDa) and L-type (≈20.2–20.8 kDa), separable by SDS-PAGE. Fourier-transform infrared (FTIR) and circular dichroism (CD) spectra reveal a secondary structure dominated by *α*-helices (52.79–72.16%), supplemented by *β*-sheets, turns, and random coils. This conformational synergy confers environmental adaptability (42, 43). The protein maintains structural stability between 60 and 80°C and pH 3.4–10; beyond these thresholds, α-helix unfolding causes functional decline. These characteristics are further illustrated through a comparison in Table 2.

**Table 2:** Comparative analysis of ferritin characteristics across biological kingdoms.

Characteristic	Sturgeon ferritin (ABLF)	Animal ferritin (e.g., Mammalian, Fish)	Plant ferritin (e.g., Soybean, Pea)	Bacterial ferritin (e.g., BFR, Dps)
Source	Liver of *Acipenser baerii* (by-product of caviar processing) (42, 87)	Vertebrates (e.g., horse spleen, human liver, fish muscle) (67, 88)	Plant seeds and tissues (e.g., soybean, pea) (25, 56, 67)	Prokaryotes (e.g., *Escherichia coli*, *Archaeoglobus fulgidus*) (8, 67)
Subunit composition	Comprises H and L subunits with molecular weights of approximately 20.8 kDa and 20.2 kD (42)	Heteropolymers of H (≈21 kDa) and L (≈19.5 kDa) chains (mammals); H-like/M subunits in lower vertebrates (67, 88)	Homopolymers of H-type subunits with 80% sequence homology; unique N-terminal extension peptide (EP) (25, 56)	BFRs: 24 H-type subunits with 12 heme groups; Dps: 12 subunits (8, 67)
Structural features	Spherical cage (outer diameter: 12.35 ± 1.22 nm, inner diameter: 5.82 ± 1.03 nm); 3-fold hydrophilic channels and 4-fold hydrophobic channels (42)	Spherical cage (12 nm outer diameter, 8 nm inner diameter); 3-fold hydrophilic channels and 4-fold hydrophobic channels (67, 88)	Spherical cage (12 nm outer diameter); 3-fold/4-fold hydrophilic channels (lined with histidines); EP stabilizes structure (25, 56)	BFRs: Heme-mediated iron release; Dps: Smaller cavity (4.5 nm) with DNA-binding capacity (8, 67)
pH stability	Stable across pH 3.4–10; dissociates at extreme pH (<2.0 or >11.0) (42, 87)	Stable at physiological pH (6.0–7.4); dissociates at extreme pH (<2.0 or >11.0) (67, 88)	Stable at neutral pH; EP enhances stability at mild acidic pH (≈4.0) (25, 56)	BFRs: Stable under alkaline conditions; Dps: Sensitive to extreme pH (8, 67)
Iron handling	Molecule stores ~Fe^3+^; H-subunits mediate ferroxidase activity; L-subunits facilitate iron mineralization. (42, 87)	H-chains: rapid Fe^2+^ oxidation; L-chains: iron mineralization and storage (67, 88)	EP acts as a secondary ferroxidase center; iron release via EP degradation (25, 56)	FRs: Heme-dependent iron release; Dps: Stores iron to protect DNA from oxidative damage (8, 22)
Encapsulation capacity	apoABLF encapsulates Ca^2+^ (≈79.77 Ca^2+^/molecule via dialysis); reversible disassembly/reassembly enables bioactive loading (42)	Encapsulates hydrophobic (e.g., curcumin) and hydrophilic (e.g., anthocyanins) compounds via pH-induced disassembly (25, 88)	Encapsulates polyphenols (e.g., EGCG) via channel expansion (urea/thermal treatment) (25, 56)	BFRs: Encapsulates metal nanoparticles (e.g., Pd); Dps: Limited capacity due to smaller cavity (8, 67)
Biocompatibility	High essential amino acid content (meets FAO/WHO standards); low immunogenicity; suitable for food/pharmaceutical applications (42)	Naturally present in animal tissues; recognized by TfR1 for cellular uptake (8, 88)	Plant-derived; non-toxic, suitable for vegetarian applications (25, 56)	May trigger immune responses in mammals; primarily used in industrial catalysis (8, 67)
Applications	Iron supplementation; functional food additives; Ca^2+^/bioactive delivery systems (42, 87)	Drug delivery (e.g., doxorubicin); iron supplementation (8, 88)	Functional foods (iron-fortified beverages); nutraceutical delivery (25, 56)	Environmental remediation (heavy metal adsorption); industrial catalysis (8, 67)

### 2.4 pH-responsive stability and functional versatility of sturgeon ferritin

Sturgeon ferritin demonstrates remarkable pH-responsive behavior, maintaining structural stability across a wide pH range (3.4–10), making it particularly suitable for oral delivery systems, functional foods, and encapsulation technologies. The protein subunits (SLF-1, SLF-2, and SLF-3) undergo reversible dissociation at extreme pH conditions while retaining the ability to reassemble at neutral pH. This unique property facilitates efficient encapsulation and targeted release of sensitive bioactive compounds, including antioxidants and therapeutic agents, throughout the gastrointestinal tract (43). Notably, the ferritin maintains stability under both gastric (pH 2–3) and intestinal (pH 6–7) conditions, effectively preventing premature degradation.

The exceptional pH tolerance enables incorporation into diverse food matrices, ranging from acidic beverages (pH 3.5–4.5) to alkaline food products (pH 8–9). Nutritionally, sturgeon ferritin provides substantial value with its high amino acid content (>600 mg/g) and balanced essential amino acid profile (SLF-1 meets FAO/WHO standards), while simultaneously serving as an effective iron-chelating agent that maintains structural integrity during fortification processes (43, 44).

The protein’s hollow nanocage architecture, combined with its pH-dependent assembly properties, allows for simultaneous encapsulation of both hydrophilic and hydrophobic bioactive compounds, including polyphenols and vitamins (44). Comparative studies by Ding. (42) demonstrate that sturgeon ferritin exhibits superior structural preservation during pH fluctuations compared to plant-derived ferritins.

## 3 Role of ferritin in iron homeostasis

Iron is an essential trace element for most terrestrial life forms. Its unique electron-donating/accepting capacity enables indispensable roles in critical biological processes, including oxygen transport, heme synthesis, DNA synthesis/repair, cellular respiration, and immune function (45). To date, two primary ferritin forms have been identified in prokaryotes: heme-containing bacterioferritins (BFRs) and canonical H-type ferritins (the latter also present in eukaryotes). Most ferritins (FTNs) adopt a typical octahedral (4–3-2) symmetric structure composed of 24 mammalian H-chain subunits (46, 47). Structural studies reveal multifunctional properties, including high stability and cellular selectivity. When exposed to extreme pH (≤2.0 or >11.0), ferritin dissociates into individual subunits; restoring neutral pH triggers their reassembly into 24-mer spherical structures with near-native secondary and tertiary conformations.

**Figure 3:**
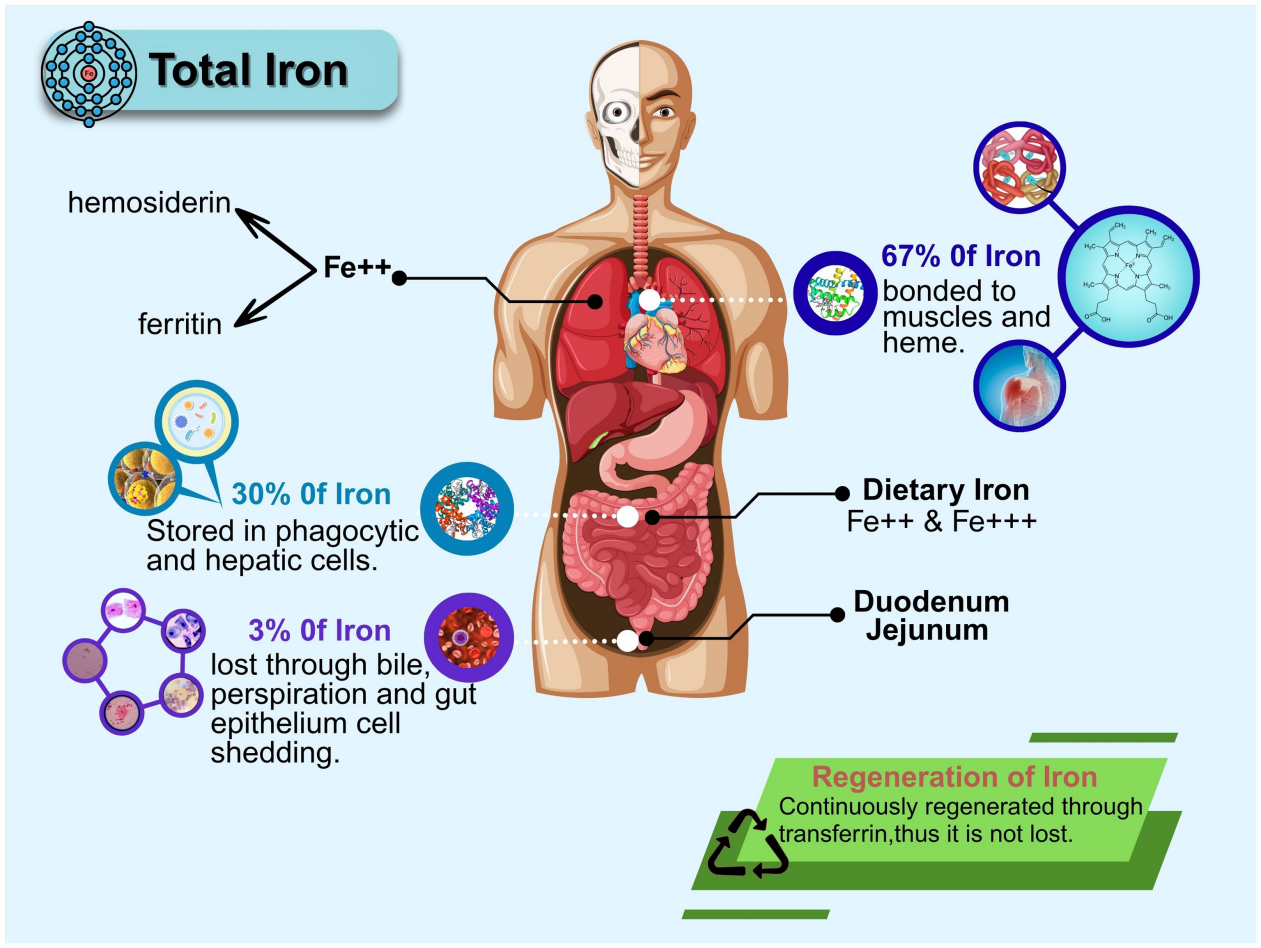
Body iron distribution and recycling pathways. This graphic depicts the overall distribution of iron within the human body and its allocation among distinct anatomical compartments. It highlights the presence of iron in various tissues and illustrates its dynamic balance.

Iron exhibits compartmentalized distribution in humans as shown in Figure 3. Ferritin acts as a regulatory iron reservoir, supplying iron ions to sustain physiological functions like heme synthesis, DNA replication, and cellular respiration. During cellular iron surplus, it stores iron in a redox-inert state. Cellular and systemic ferritin levels serve not only as key indicators of iron status but also as biomarkers for immune disorders, malignancies, and inflammatory processes (48–51). By precisely regulating iron storage/release, ferritin maintains optimal iron balance in cells and tissues. This process is co-ordinately controlled at transcriptional and translational levels by iron response elements (IREs) and iron regulatory proteins (IRPs) (30).

From a human health perspective, iron overload and deficiency are prevalent worldwide and closely linked to genetic factors. Dysregulated iron metabolism represents a major global health challenge (52). Systemic iron status modulates intestinal iron absorption via ferritin levels: elevated ferritin signals iron sufficiency, downregulating iron absorption pathways to prevent overload.

## 4 Multifunctional applications of ferritin

### 4.1 Applications in food engineering and nutrition

#### 4.1.1 Intervention in iron deficiency anemia and iron fortification

Iron deficiency anemia (IDA) is the most prevalent global nutritional disorder, primarily caused by inadequate dietary iron intake and low iron bioavailability. While ferrous sulfate and ferrous gluconate serve as iron supplements, they induce adverse effects such as constipation, diarrhea, and growth retardation (53). Legume seed ferritin is recognized as a high-quality natural dietary iron source, with 90% of its iron stored in ferritin form. A standardized study assessed iron absorption efficiency from purified soybean ferritin using a test meal (bagel with cream cheese and apple juice) containing 1 μCi ^59^Fe per serving (as FeSO₄ or phosphate-reconstituted iron-free soybean ferritin). Results demonstrated comparable whole-body iron absorption between soybean ferritin and ferrous sulfate, confirming its high iron absorption rate and offering a novel plant-based strategy for iron-deficient populations (54). The plant ferritin shell effectively prevents iron ions from reacting with food components like oxalate and tannic acid.

As a natural iron carrier, sturgeon liver ferritin exhibits high bioavailability and low gastrointestinal irritation, making it a viable alternative to traditional iron salts for food fortification. Its amino acid profile complies with FAO/WHO standards, providing both iron and high-quality protein. Inductively coupled plasma mass spectrometry (ICP-MS) confirmed that apoferritin binds ~79.77 calcium ions per molecule, enabling its use as an ideal carrier for minerals and bioactives (e.g., vitamins, polyphenols). Co-encapsulation of vitamin C and iron leverages antioxidant synergy to simultaneously enhance iron absorption and product stability (42).

#### 4.1.2 Encapsulation and stabilization of bioactive nutrients

Bioactive compounds—such as proanthocyanidins (PAs), lutein, *β*-carotene, curcumin, rutin, and cyanidin-3-glucoside (C3G)—possess antioxidant, anticancer, and anti-inflammatory properties but are highly sensitive to pH, light, heat, and oxygen, leading to degradation and isomerization (55). Ferritin’s reversible self-assembly, exceptional thermostability, and intrinsic nanocavity render it an ideal delivery vehicle. Studies show that ferritin nanoencapsulation significantly enhances the stability of these compounds versus free forms (*p* < 0.01) and improves water solubility of lipophilic molecules (25).

Three key factors contribute to the enhanced stability of bioactive nutrients within ferritin nanocages, with these mechanisms offering multifunctional benefits in food nutrition: the protein shell acts as a physical barrier isolating cavity-encapsulated molecules from aqueous-phase pro-oxidants while shielding them from temperature and light exposure; functional groups such as cysteine residues exert antioxidant effects by chelating transition metals or scavenging free radicals; and ferritin forms molecular complexes with nutrients through hydrogen bonding, van der Waals forces, and hydrophobic interactions, thereby protecting bioactive components from degradation. Beyond improving stability and water solubility, ferritin encapsulation may enhance intracellular nutrient absorption efficiency, while ferritin-templated nanoparticles demonstrate significant potential for heavy metal ion detection in food safety applications.

#### 4.1.3 Food preservation, quality modulation, and shelf-life extension

Ferritin’s unique nanocage structure (10–12 nm diameter) and multifunctionality offer significant potential in food preservation as listed in Table 1. As an efficient carrier, it encapsulates hydrophobic or hydrophilic compounds (e.g., resveratrol, chlorogenic acid), markedly improving their stability. Dextran-ferritin nanoparticles stabilize multiple emulsion systems to co-deliver resveratrol, chlorogenic acid, and astaxanthin, enhancing nutrient retention and antioxidant activity in freeze-dried apple slices (10). Moreover, ferritin’s exceptional iron-storage capacity (molecular mass: 474 kDa) enables its effective use as a nutritional iron fortificant, offering superior bioavailability while minimizing gastrointestinal irritation.

Ferritin demonstrates notable antioxidant and antimicrobial properties for quality control. Amino acid residues (e.g., glutamate, phenylalanine) scavenge free radicals, suppressing lipid oxidation and enzymatic browning. Ferritin-based emulsions reduce moisture absorption in freeze-dried apples by 30% while elevating DPPH radical scavenging to 85%. Critically, ferritin forms protective coatings on food surfaces, improving texture (e.g., 20% hardness increase) and reducing water activity (a < sub > w</sub > < 0.15) to extend shelf life. As a Pickering emulsifier, it stabilizes oil-in-water emulsions for controlled bioactive release. W1/O/W2 double emulsions prepared with dextran-ferritin nanoparticles exhibit uniform droplet distribution and enhance processing stability of actives (10). These attributes position ferritin as a key tool in functional food development and preservation.

Ferritin shows high promise for fresh-cut fruit preservation. Dextran-ferritin-resveratrol nanoparticles significantly improve freeze-dried apple slice quality: after 60-min emulsion treatment, brightness (L*) increases from 71.69 to 80.82, hardness and antioxidant activity (85% DPPH scavenging) rise concurrently, while total sugars, acids, and vitamin C are preserved (10). Sturgeon liver ferritin with analogous nanocage structure and antioxidant amino acids (e.g., glutamate, phenylalanine) holds theoretical potential for broader fruit preservation but requires matrix-specific efficacy validation and extraction optimization.

### 4.2 Applications as nanocarriers

#### 4.2.1 Encapsulation techniques and efficiency

Ferritin nanocages serve as highly efficient nanocarriers in food industry applications, extensively utilized to encapsulate as deliberated in.

Table 3 bioactive compounds such as proanthocyanidins, rutin, curcumin, anthocyanins, and *β*-carotene (31, 56–60). Ferritin-based encapsulation significantly enhances the stability, cellular uptake efficiency, and water solubility of these functional components (e.g., epigallocatechin gallate). The nanocages accommodate diverse nutrients including vitamins, minerals, antioxidants, and *ω*-3 fatty acids shielding them from oxidation, degradation, and interactions with other food constituents during storage and processing. Flavor compounds (e.g., natural extracts, essences) can also be encapsulated within ferritin nanocages. Bioactive compounds have gained widespread application in health promotion and disease prevention. However, their practical utility is often limited by environmental instability, poor solubility, low bioavailability, and nonspecific delivery. Among various nanoscale delivery systems, protein-based carriers have emerged as particularly promising vehicles for bioactive encapsulation and targeted delivery, owing to their superior biocompatibility and functional versatility (17, 61).

**Table 3:** Encapsulation efficiency and stability of bioactive compounds in ferritin.

Bioactive compound	Increase in stability (%)	Change in water solubility (Fold Increase)	Cellular absorption efficiency (Relative to Free Form)
Procyanidins	50%	2-fold	1.5-fold
Rutin	40%	1.8-fold	1.3-fold
Curcumin	60%	3-fold	2-fold
Anthocyanin	45%	2.2-fold	1.4-fold
β-Carotene	55%	2.5-fold	1.6-fold

#### 4.2.2 Release kinetics and carrier diversity

A key advantage of ferritin nanocages is their capacity for controlled release. Modulating cage properties (e.g., structural integrity, surface chemistry) enables precise tuning of release kinetics. For oral delivery in functional foods or supplements, ferritin’s biological behavior requires critical evaluation: structural stability in the gastrointestinal tract is essential for intact transport and absorption of actives, yet studies indicate potential complete degradation by digestive enzymes (e.g., pepsin, trypsin) before reaching target organs like the intestine (62).

Peptides, as self-assembling units, form protein nanotubes offering a viable alternative to carbon nanotubes for delivering iron and other bioactives in functional foods (63). Research on hydrophilic compound delivery (e.g., lycopene) remains limited. *α*-Lactalbumin (α-La), a milk-derived protein rich in essential amino acids, self-assembles into nanotubes with calcium assistance, exhibiting enhanced bioavailability, prolonged intestinal retention, and superior cellular uptake (64). Chen et al. (17) demonstrated that α-La-based dairy delivery systems significantly improved lycopene absorption in the small intestine while optimizing texture, stability, and viscosity in liquid foods, establishing a novel route for iron and bioactive release (65).

Ferritin-derived crystalline nanoparticles (“nanocrystals”) show promise in nano-fortification. Liu et al. (66) and Zhang et al. (67) evaluated starch nanocrystals and nanoparticles for pH-responsive oral tannic acid delivery, confirming their antimicrobial, antioxidant, and biocompatible properties. Starch nanocrystals exhibit excellent biocompatibility and low cytotoxicity. Hydroxyethyl starch, propyl starch, oxidized starch, hydroxybutyrate, and succinylated starch serve as novel carriers for iron delivery in functional foods (68). Ferritin-based nanocarriers thus offer flexible, efficient solutions to enhance flavor, functionality, and nutritional value while ensuring stability and safety.

#### 4.2.3 Current status of nanotechnology in food industry and ferritin’s role

Food nanotechnology leverages nanoparticles of varying sizes, compositions, and morphologies to drive innovation. Owing to their speed, sensitivity, and efficiency, nanosensors detect contaminants, pathogens, allergens, and nutrients in food science (69). Combined with traditional polymers, nanomaterials enhance durability, abrasion resistance, and barrier properties against moisture, light, vapor, and gasses. Nanocomposites formed by integrating polymers with nanofillers—improve packaging performance. Nanocoatings are the most common barrier-enhancement technology, applying thin films to create mass-transfer barriers (70). Clay nanocomposites reduce O₂/CO₂ permeability by 80–90%. Nanoencapsulation extends shelf life while optimizing physical properties of foods and packaging materials. Key applications include coatings, packaging, and nanobiosensors for detecting food-spoiling bacteria and chemicals (8, 71).

**Figure 4:**
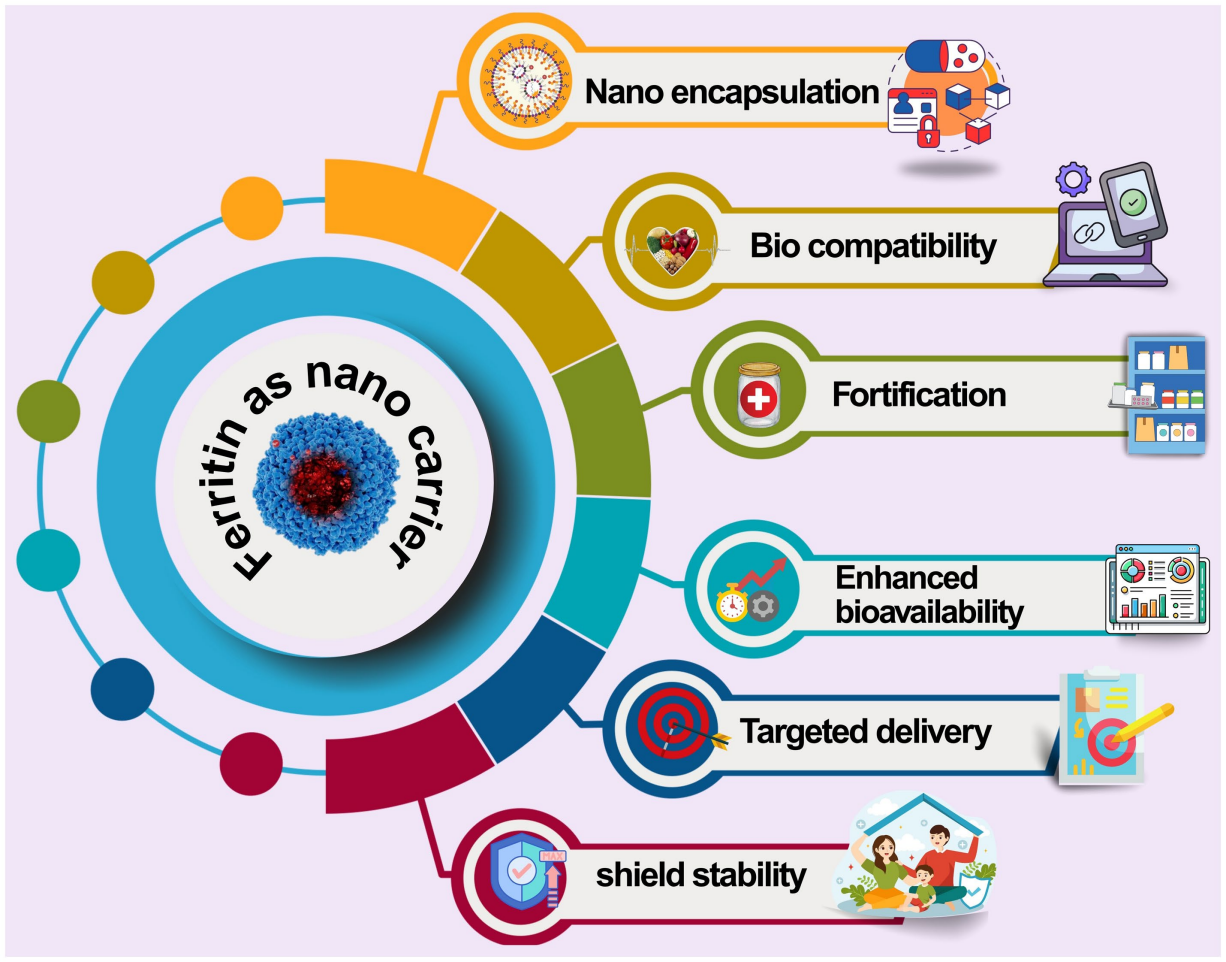
Ferritin nanocarriers for bioactive encapsulation in food. Ferritin encapsulates bioactive compounds, enhancing their stability and bioavailability. This approach enables controlled nutrient delivery and can improve the organoleptic properties and nutritional profile of food products. Ferritin-based nanocarriers represent a promising strategy for developing functional foods.

As a natural protein nanocarrier as shown in Figure 4 ferritin’s biocompatibility, reversible assembly, and protective encapsulation offer unique advantages in shelf-life extension (especially for perishable, browning-prone fresh-cut fruits) and stable nutrient delivery, positioning it as a versatile platform in food nanotechnology.

#### 4.2.4 Potential risks and challenges of nanotechnology

Nanoparticles in food science may pose unpredictable hazards and potential toxicity to humans, animals, and the environment. Currently, the U. S. Environmental Protection Agency (EPA) and Food and Drug Administration (FDA) lack standardized regulations for nanomaterial use. Unique physicochemical properties of nanomaterials complicate toxicological assessments due to differential toxicity profiles. As particle size decreases, toxicity increases; nanoparticles can penetrate biological barriers into cells and organs unachievable by conventional particles (72). Although ferritin, as a natural biomolecule, exhibits relatively higher biosafety, long-term intake effects, potential immunogenicity, and behavior in complex food matrices require careful investigation.

### 4.3 Biomedical applications

**Figure 5:**
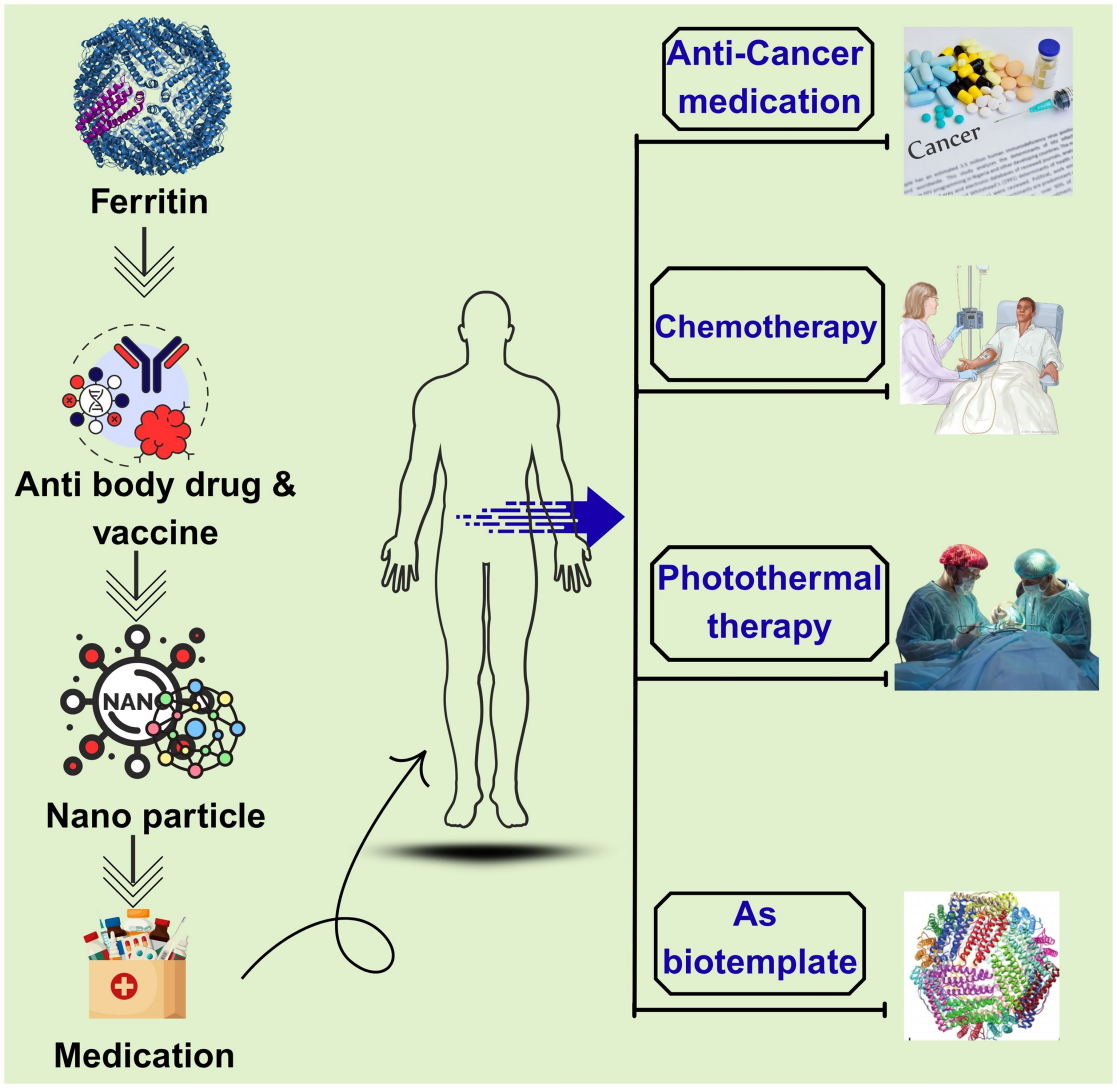
Therapeutic applications of ferritin nanoparticles in medicine. The ferritin structure serves as a versatile platform for vaccine and antibody delivery, enabling its use as a nanocarrier in diverse therapeutic interventions. Key applications include bio-templating, photothermal therapy, and chemotherapy, with notable potential in cancer treatment and other human health advancements.

Ferritin nanocages serve as versatile biological templates for chemotherapy drug encapsulation, photothermal therapy, and *in vivo* imaging. Beyond drug delivery, loading with metal nanoparticles and dye molecules enables optical and magnetic resonance imaging (MRI), providing critical support for early cancer detection, diagnostic subtyping, treatment monitoring, and prognostic assessment as depicted in Figure 5. The 12-nm size of sturgeon liver ferritin facilitates tumor targeting via the enhanced permeability and retention (EPR) effect, elevating intratumoral drug concentration while reducing systemic toxicity in murine models.

Digestive stability of ferritin in the gastrointestinal tract critically determines its efficacy for nutrient and drug delivery. Nanoparticles failing to withstand digestive conditions lose therapeutic value (25). Ferritin’s unique advantages include: its protein cage shields against complexation by dietary factors (e.g., phytic acid, oxalate, tannins); natural seed coat protection confers gastric stability; and its slow-release iron core fundamentally differs from conventional iron supplements (inorganic/organic salts) and natural chelates (e.g., Fe(III)-phytate) in physicochemical behavior (73).

The ferritin cavity functions as a nanoreactor for precise synthesis of functional materials (e.g., magnetic Fe₃O₄). Polyethylene glycol (PEG) surface modification extends *in vivo* circulation half-life, while quantum dot hybrids combine structural stability with fluorescence for advanced bioimaging. Compared to conventional synthesis, ferritin-templated technology confers superior monodispersity and functional programmability to nanomaterials (30, 43, 74).

## 5 Challenges and future directions in ferritin research

### 5.1 Controversial findings on iron bioavailability

While ferritin is recognized as an effective dietary iron source for iron-deficient populations, its bioavailability remains a subject of debate. Conflicting results have been reported, particularly regarding iron absorption from plant-based ferritins such as soybean ferritin. Nonetheless, several studies have demonstrated that ferritin-bound iron is bioavailable and can contribute meaningfully to alleviating iron deficiency. For instance, the bioavailability of iron from pea ferritin has been validated in dietary trials, and ferritin-delivered iron has been shown to be comparable in absorption to traditional iron salts like ferrous sulfate, especially in non-anemic individuals (75, 76).

The variability in outcomes may be attributed to several influencing factors, including physiological conditions, dietary composition, and ferritin origin or structural differences (76). Radioisotope-based studies, which are commonly employed to assess iron absorption, have revealed inconsistencies. Early *in vitro* and *in vivo* experiments using radiolabeled ferritin reported contrasting results particularly with legume seed ferritin, where nearly 90% of seed iron was stored as ferritin, yet showed limited bioavailability in exogenously labeled studies (77). A major limitation stems from the challenges of uniform radiolabeling in vivo, which can result in incomplete tagging of ferritin iron and lead to differential absorption of unlabeled iron. These methodological discrepancies complicate accurate evaluation of ferritin’s true bioavailability. Given its nutritional importance, especially for combating global iron deficiency, resolving these inconsistencies is critical. Future research should prioritize advanced radiolabeling and stable isotope techniques, structural modification of the ferritin shell through genetic engineering, and the development of encapsulation approaches to more precisely track and enhance ferritin iron absorption.

### 5.2 Dual role as inflammation biomarker and degradation mechanisms

Ferritin functions not only as an iron status biomarker but also as a key indicator of inflammation. Altered glycosylation patterns during inflammatory and oncologic processes offer novel avenues for early diagnosis (62, 78). Inflammatory cytokines upregulate H-and L-subunit expression at transcriptional and translational levels (79). When iron demand surges or deficiency occurs, stored iron must be mobilized primarily via lysosomal protease-mediated ferritin degradation (80). Autophagy mechanisms participate in this process, with recent studies identifying nuclear receptor coactivator 4 (NCOA4) as a cargo receptor mediating targeted lysosomal degradation of ferritin, termed ferritinophagy (89). Progressive elevation of ferritin and other inflammatory markers consistently correlates with disease severity and mortality (81–84).

### 5.3 Future research priorities

Future ferritin research must focus on several critical areas. The primary task involves deepening mechanistic understanding of ferritin structural stability, particularly its dynamic behavior in complex food matrices and during digestion, while systematically elucidating molecular pathways of iron release and absorption to resolve controversies surrounding iron bioavailability.

For application bottlenecks in food engineering, innovative uses in fresh-cut fruit shelf-life extension should be prioritized, including: mechanistic investigation into how ferritin-encapsulated antioxidants (e.g., ascorbate, polyphenols) inhibit enzymatic browning and oxidative deterioration; development of smart coatings or targeted delivery systems (incorporating sturgeon liver ferritin) for controlled release of actives on cut surfaces; comprehensive evaluation of ferritin carriers’ effects on sensory quality (color/texture/flavor), microbial safety, and retention of key nutrients (e.g., vitamin C, polyphenols) in fresh-cut produce; and tackling stability challenges in high-water-activity, complex-matrix food systems.

Concurrently, systematic assessment of long-term intake safety including potential immunogenicity, metabolic pathways, and chronic toxicity requires strengthening. Industrially, scaling production processes (e.g., efficient sturgeon ferritin extraction/purification) is urgently needed to significantly reduce costs and increase yields. Parallel efforts should expand ferritin’s cross-disciplinary applications in precision nutrition delivery, tumor-targeted therapy, inflammatory disease intervention, and stimuli-responsive materials.

Current clinical translation faces dual challenges: prohibitive extraction costs and insufficient long-term safety evidence. Future advancements necessitate breakthroughs in scalable production technologies and establishing an integrated “basic research → technology development → industrial application” framework through interdisciplinary collaboration to fully unlock ferritins potential across food engineering, biomedicine, and materials science.

## 6 Conclusion

Ferritin emerges as a versatile interdisciplinary platform at the intersection of food science, nanotechnology, and biomedicine. Its inherent biocompatibility, dynamic nanocage architecture, and encapsulation capabilities position it as a valuable tool for addressing critical challenges in nutrient delivery, diagnostics, and food preservation. Among its variants, sturgeon liver ferritin is particularly notable for its superior physicochemical stability and iron-loading capacity, making it well-suited for application in acidic and low-temperature food systems. However, challenges such as species-specific variability, limited digestive stability, and inconsistent bioavailability remain unresolved. Advancing this field will require targeted strategies including genetic engineering, material modification, and rigorous clinical validation. Cross-disciplinary collaboration will be essential to unlock ferritin’s full potential in promoting food security and improving public health.
